# Evaluating the cost-effectiveness of levofloxacin therapy for household contacts of multidrug-resistant tuberculosis in Vietnam

**DOI:** 10.1016/j.lanwpc.2025.101666

**Published:** 2025-08-08

**Authors:** Tasnim Hasan, Nguyen Thu Anh, Nguyen Binh Hoa, Nguyen Viet Nhung, H. Manisha Yapa, Stephen M. Graham, Ben J. Marais, Guy B. Marks, Tom Lung, Greg J. Fox

**Affiliations:** a NHMRC Clinical Trials Centre, Faculty of Medicine and Health, University of Sydney, Sydney, Australia; b The Sydney Infectious Diseases Institute (Sydney ID), The University of Sydney, Sydney, Australia; c The University of Sydney Vietnam Institute, University of Sydney, Ho Chi Minh City, Vietnam; d Woolcock Institute of Medical Research, Hanoi, Vietnam; e National Lung Hospital, Hanoi, Vietnam; f University Medical Centre, Ho Chi Minh, Vietnam; g Melbourne Children’s Global Health, University of Melbourne Department of Paediatrics and Murdoch Children’s Research Institute, Royal Children’s Hospital, Melbourne, Australia; h Burnet Institute, Melbourne, Australia; i School of Public Health, Faculty of Health and Medicine, University of Sydney, Sydney, Australia; j The George Institute for Global Health, University of New South Wales, Sydney, Australia; k Royal Prince Alfred Hospital, Sydney Local Health District, Camperdown, Australia

**Keywords:** Levofloxacin, Drug-resistant tuberculosis, Cost-effectiveness, Household contacts

## Abstract

**Background:**

Multidrug-resistant tuberculosis (TB) threatens global TB control, on account of poor treatment outcomes, high treatment toxicity and costs. Recent trials demonstrated the effectiveness of six-months of levofloxacin (6Lfx) to prevent TB disease among high-risk contacts. However, the cost-effectiveness of this strategy has not previously been evaluated.

**Methods:**

The VQUIN study was a double-blinded randomised control trial in Vietnam assessing the effectiveness of 6Lfx in household contacts of multidrug resistant/rifampicin resistant TB (MDR/RR-TB) to prevent progression to TB disease. Incorporating in-trial costs and effectiveness outcomes from the VQUIN trial, we developed a closed cohort, decision-analytic Markov model to assess the cost effectiveness of 6Lfx versus placebo in a cohort exposed to MDR/RR-TB in Vietnam.

**Findings:**

Over a 20-year time horizon, the provision of 6Lfx preventative therapy to household contacts of people infected with MDR/RR-TB was found to gain a total of 40.1 QALYs per 1000 population and save US$23,145 per 1000 population, indicating the strategy was cost saving. MDR/RR-TB cases averted over 20 years was 19.9 per 1000 population treated with 6Lfx, and the number of deaths averted was 3.2 per 1000 people treated.

**Interpretation:**

6Lfx therapy is a cost-saving strategy to reduce the incidence of active disease in household contacts of MDR/RR-TB in a resource-limited setting.

**Funding:**

10.13039/501100000925National Health and Medical Research Council Project Grant (#1081443). GJF was supported by a NHMRC Leadership Fellowship (Level 1) (#2007920).

**Research in context**

**Evidence before this study**

Two recent randomised trials—the VQUIN and TB-CHAMP studies—showed that six months of daily levofloxacin (6Lfx) as tuberculosis preventive treatment (TPT) reduced the incidence of tuberculosis (TB) among household contacts of people with multidrug-or rifampicin-resistant tuberculosis (MDR/RR-TB). These were the first trials to establish the efficacy of TPT for MDR/RR-TB contacts. However, cost assessment is crucial for 6Lfx to be incorporated as TPT into national TB programs in resource-limited settings.

To evaluate the literature discussing cost-effectiveness of TPT, we systematically searched MEDLINE and Embase. Several studies evaluated the cost-effectiveness of TPT for drug susceptible (DS)-TB, and only one study for MDR/RR-TB. However, this single study modelled hypothetical efficacy data, and applied cost parameters from a high-income setting. No studies were available which used trial outcomes to estimate cost-effectiveness of treating contacts of people with MDR/RR-TB, in a high TB prevalence setting.

**Added value of this study**

This study investigated the cost-effectiveness of 6Lfx versus no TPT in the prevention of developing MDR/RR-TB in a high-prevalence setting. We developed a closed cohort, decision-analytic Markov model that followed a single exposure cohort in annual cycles over a time horizon of 20-years–from a health system perspective. The analysis applied in-trial costs from the VQUIN trial in Vietnam, a high TB incidence setting, MDR/RR-TB for transition probabilities and cost-estimates. This is the only study assessing the cost-effectiveness of MDR/RR-TB TPT using trial data. Compared to placebo, 6Lfx resulted in a gain of over 40 quality associated life years (QALYs) per 1000 adult (>18 years) MDR/RR-TB contacts treated with 6Lfx. The strategy was cost-saving. 6Lfx preventative therapy averted 19.9 cases of MDR/RR-TB and averted 3.2 cases of death per 1000 people treated.

**Implications of all the available evidence**

Our study findings demonstrate that levofloxacin TPT is cost-saving for national TB programs in high TB incidence settings. We demonstrate savings in cost, an increase in QALYs, reduced MDR/RR-TB cases and lower TB related mortality. These findings support the use of 6Lfx TPT for individuals at high risk of developing MDR/RR-TB in resource-limited settings.

## Introduction

In 2023 an estimated 400,000 people developed multi-drug resistant/rifampicin-resistant (MDR/RR) tuberculosis (TB).^1^ However, only 175, 000 were diagnosed,^1^ with those undiagnosed remaining an ongoing transmission risk for close contacts.^2^ Furthermore, MDR/RR-TB is associated with catastrophic costs to both individuals and society.^3^

Tuberculosis preventative therapy (TPT) has been shown to reduce the incidence of TB disease among high-risk contacts.^4^ While several regimens are available to treat TBI in those who are contacts of drug-susceptible (DS)-TB,^4^ the options for contacts of those diagnosed with MDR/RR-TB remains limited. Two recent clinical trials found that a six-month course of daily levofloxacin (6LFx) was associated with a reduction in the incidence of TB disease among adult and child household contacts of individuals diagnosed with MDR/RR-TB.^5^^,^^6^ Effective treatment for tuberculosis infection (TBI) is critical in reducing the health and economic impacts of MDR/RR-TB among high-risk individuals.

The VQUIN MDR/RR-TB study was a randomised placebo-controlled trial conducted in Vietnam, which investigated the efficacy of 6Lfx therapy provided to household contacts of those diagnosed with MDR/RR-TB. TB-CHAMP was a trial conducted in South Africa, assessing the efficacy of 6Lfx therapy in preventing TB in MDR/RR-TB contacts who were children.^5^ The probability of cure with 6Lfx was determined to be 0.6, from a Bayesian analysis using both VQUIN and TB-CHAMP data.^7^ TPT with 6Lfx was shown to be safe and did not lead to acquired drug resistance among those who developed incident TB.^6^

The implementation of 6Lfx as TPT into national policy will require considerations of programmatic costs in resource-limited settings. One consideration in the introduction is the cost-effectiveness of the 6Lfx strategy compared to no treatment. To date, no studies have evaluated the cost-effectiveness of TPT for MDR/RR-TB following the publication of data from randomised trials.

This study aimed to evaluate the cost-effectiveness of 6Lfx, in Vietnam, compared to placebo, provided for individuals who are household contacts of those who are confirmed to be infected with MDR/RR-TB.

## Methods

We developed a closed cohort, decision-analytic Markov model, from a health system perspective, that followed a single cohort in annual cycles over a time horizon of 20 years. Individuals entered the model at an average age of 40 years. Outcomes included cost per quality-adjusted life-year (QALY) gained and TB cases averted. Costs were adjusted to 2023 US dollars. Future costs and outcomes were discounted at 3%.^8^ The Consolidated Health Economic Evaluation Reporting Standards (CHEERS) checklist was used to guide the methodology of this project (Supplementary Table S1).^9^

### Setting

Vietnam is a lower-middle income country in Southeast Asia with a population of over 100 million.^10^ More than 100,000 TB cases are reported yearly, of which approximately 5% are MDR/RR-TB.^6^^,^^11^ Screening, diagnosis and treatment are provided without cost to patients by the National TB Program (NTP) in all of Vietnam’s 63 provinces.

### VQUIN trial of levofloxacin preventive therapy

The VQUIN study was a double-blind randomised control trial across ten provinces in Vietnam. Between 2016 and 2019, participants were randomised to either levofloxacin or placebo once daily for six months.^6^ Levofloxacin was prescribed orally, at 10–15 mg per kilogram of body weight for adults and 15–20 mg per kilogram for children, with a maximum dose of 750 mg daily. The primary objective was to evaluate the cost-effectiveness of levofloxacin given for 6 months, compared to placebo, at preventing TB disease among household contacts of people with MDR/RR-TB. A total of 2041 participants were randomised to levofloxacin or identical placebo. Six cases (0.6%) of confirmed TB occurred in the 6Lfx group and 11 (1.1%) in the placebo group, with no difference in severe adverse events and no acquired fluroquinolone resistance detected (for those who developed TB disease), between the two groups after 54 weeks of follow up.

### Description of the model

**Fig. 1:**
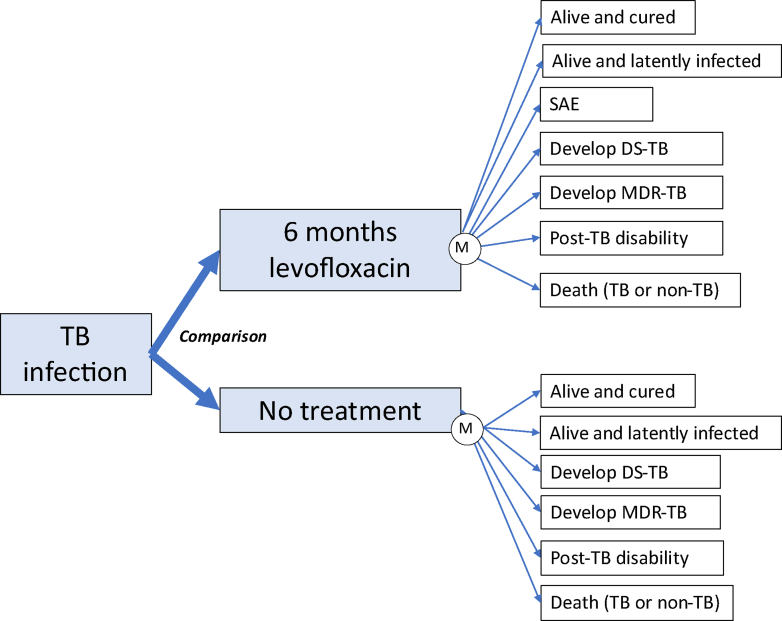
Decision analysis tree for 6Lfx versus not treatment for household contacts of MDR/RR-TB cases. DS-TB drug susceptible tuberculosis, QALY Quality adjusted life years, MDR-TB multi-drug resistant tuberculosis, SAE serious adverse event, TB tuberculosis.

A Markov decision analysis model evaluated the cost-effectiveness of providing 6Lfx therapy to household contacts of confirmed MDR/RR-TB compared to no TPT. Fig. 1 describes the model developed. Mortality was calculated over a 20-year period, by applying standard life tables to a population aged 40 in the first year of the study. TB infection was determined by a positive tuberculin skin test or considered if the household contact was immunosuppressed. After TB disease was excluded based upon clinical assessment, chest radiography and sputum sampling as required, individuals were given 6Lfx or placebo, with monitoring for development of TB disease. Following this, individuals could enter six different and mutually exclusive health states: 1) Cured of TB and remained alive and healthy, 2) Remaining alive but remained infected with TB, 3) Developing MDR/RR-TB, 4) Developing DS-TB 5) Developing persisting disability after a diagnosis and treatment for TB and 6) Death (due to TB or other causes). For those entering the 6Lfx treatment arm, an additional health state could be entered in the first year of the model only: developing a severe adverse event from 6Lfx treatment. For the purpose of this model, it was assumed that individuals could not be re-infected with a new TB strain or develop TB disease.

### Transition probabilities

Annual transition probabilities, costs and utility for each transitional state are presented in Table 1. The Markov decision tree and model is available in Supplementary Figs. S1 and S2. The probabilities for cure, TBI and developing TB were based on VQUIN trial data. From the trial data, the probability of developing TB disease was 0.011 in household contacts who were not treated with 6Lfx, over two years. Of individuals who developed TB in the placebo and intervention arm, 75% developed MDR/RR-TB.^6^ After two years, the long-term risk of developing MDR/RR-TB in household contacts was estimated to be 0.001 (hazard ratio 2.81 (95% CI 0.89–8.83)).^12^ The probabilities for TB outcomes (cure, failure, death) were based on published literature and programmatic data from the Vietnamese NTP.^6^^,^^11^^,^^19^ The probability of cure with 6Lfx was 0.6, using the Bayesian analysis combing both VQUIN and TB-CHAMP data (hazard ratio 0.41, 95% CI 0.18–0.93).^7^ The age adjusted probability of mortality was applied over 20 years for those not infected with TB using Vietnamese life tables (2020).^14^ The risk of mortality for those with chronic TB disability was estimated at 0.026 (95% CI 0.22–0.31) per year for the first five years after infection, based on follow up data available from the VQUIN trial.^13^

**Table 1:** Estimates of probabilities, utilities and costs used in cost-effectiveness model.

Parameter	Estimate	Distribution used	References
Time horizon	20 years		
Discount rate	0.03		
Age of entry into model	40		
Probability of cure with 6Lfx	0.6	Beta	^6^^,^^7^
Probability of developing MDR/RR-TB in household contacts with 6Lfx (first two years)	0.00165	Beta	^6^
Probability of developing MDR/RR-TB in household contacts without 6Lfx (first two years)	0.00413	Beta	^6^
Probability of developing DS-TB in household contacts with 6Lfx (first two years)	0.00055	Beta	^6^
Probability of developing DS-TB in household contacts without 6Lfx (first two years)	0.00138	Beta	^6^
Probability of developing MDR/RR-TB in household contacts with 6Lfx (after two years)	0.00075	Nil	^12^
Probability of developing MDR/RR-TB in household contacts without 6Lfx (after two years)	0.0003	Nil	^12^
Probability of developing DS-TB in household contacts with 6Lfx (after two years)	0.00025	Nil	^12^
Probability of developing DS-TB in household contacts without 6Lfx (after two years)	0.0001	Nil	^12^
Probability of severe adverse event during treatment	0.028	Beta	^6^
Probability of death with severe adverse event during treatment	0	Beta	^6^
Outcomes during treatment for drug-susceptible TB			
Probability of DS-TB treatment failure	0.07	Beta	^11^
Probability of death while on DS-TB treatment	0.02	Beta	^11^
Outcomes during treatment for MDR/RR-TB			
Probability of MDR/RR-TB treatment failure	0.31	Beta	^11^
Probability of death while on MDR/RR-TB treatment	0.09	Beta	^11^
Probability of death with chronic TB (first 5 years)	0.02	Beta	^13^
Probability of death due to causes other than tuberculosis	Life table	Beta	^14^
Quality of life parameters			
QALY of being alive	Age-specific utility values	Beta	^15^
QALY of death	0		
QALY with DS-TB	0.58	Beta	^16^
QALY with MDR/RR-TB	0.51	Beta	^17^
QALY of post-TB lung disease	0.88	Beta	^18^
Cost parameters			
Cost of screening for TB	US$2.4	Gamma	^6^^,^^19^
Cost of 6Lfx	US$42	Gamma	^6^^,^^19^
Cost of a severe adverse event while on 6Lfx	US$260	Gamma	^6^^,^^19^
Cost of DS-TB treatment	US$145	Gamma	^20^
Cost of MDR/RR-TB treatment	US$1353	Gamma	^21^
Cost of post-TB lung disease	US$2800	Gamma	^22^

6Lfx, 6 months of daily levofloxacin; DS-TB, drug susceptible tuberculosis; QALY, Quality adjusted life years; MDR/RR-TB, multi-drug resistant tuberculosis/rifampicin resistant; TB, tuberculosis; US, United States.

### Utilities

Age specific utility values derived from the general Vietnamese population were used for health-related quality of life from the age of 40–60 years.^15^ The QALY associated with having MDR/RR-TB,^17^ TB^16^ and chronic post-TB lung disease^18^ were based on published global data.

### Costs

Estimated costs for drugs (6Lfx), screening for TB (using clinical assessment, chest radiography and sputum sampling as required), adverse events, monitoring for adverse events and hospitalisation costs were derived from cost norms provided by the Vietnamese NTP. The cost of managing adverse events included hospitalisation costs, costs of investigations and health care personnel costs. In The VQUIN trial, the average length of stay for a severe (grade 3 or 4) adverse event was 5.3 days. Costs of WHO-recommended standardised regimens for treating MDR/RR-TB^21^ and the estimated yearly cost of post-TB sequela was derived from studies in Southeast Asian settings.^22^ Costs resulting from post-TB disabilities were based on published estimates and included health care associated disability for pulmonary and other health impacts as well as loss of household income.^22^ Costs were estimated in 2023 US dollars. The incremental cost-effectiveness ratio (ICER) for each incremental QALY averted was calculated. The willingness-to-pay threshold was set at US$4500, which is approximately equivalent to the Vietnamese GDP per capita.^10^ As this analysis was from a health system costs perspective, out-of-pocket expenditures for people with TB and their contacts were not captured.

### Sensitivity analyses

One-way sensitivity analyses were evaluated for uncertainties in the model including uncertainties for the probability of developing MDR/RR-TB among contacts, the probability of effective prevention with 6Lfx, probabilities of TB treatment outcomes. Uncertainties for each QALY and cost values were also factored into the sensitivity analyses. Estimates for programmatic costs for MDR/RR-TB, as well as costs and quality of life for post-TB lung disease, vary considerably in the literature. Hence, wide ranges were used for each parameter to evaluate the impact of data uncertainties. The upper and lower limits of each parameter is presented in Supplementary Table S2.

Probabilistic sensitivity analyses (PSA) were performed using 1000 iterations for all parameters. Uncertainty for each parameter was assigned using a beta distribution for probabilities and utilities (as utilities and probabilities are between 0 and 1) and a gamma distribution for costs (as costs are non-negative and continuous) (Table 1).

The model was built and analysed using TreeAge ProTM (TreeAge Software Inc, Williamstown, Massachusetts, USA).

### Ethics approval

Ethical approval was obtained during the initial VQUIN trial: Human Research Ethics Committee of the University of Sydney (2014/929) and by the Institutional Review Board at the Ministry of Health Vietnam (4640/QD-BYT and 94/CN-HDDD).

### Role of funding source

The funding body did not have any role in study design, data collection, data analysis, interpretation or writing of the report.

## Results

Over a 20-year time horizon, the provision of 6Lfx to individuals at high risk of MDR/RR-TB resulted in a gain of 40.1 (95% CI 20.2, 178.7) QALYs per 1000 population and saved $23,145 (95% CI $5787; $43,604) per 1000 population, compared to no treatment. The costs were lower and QALYs greater for 6Lfx compared to no treatment (Table 2). The number of MDR/RR-TB cases averted over 20 years was 19.9 per 1000 population (95% CI 19.8, 20.0), and 3.2 deaths (95% CI 3.1, 3.3) were averted per 1000 population.

**Table 2:** The cost-effectiveness of 6 months of levofloxacin therapy versus placebo in household contacts of MDR/RR-TB cases (20 years).

	Total cost (US$ (95% CI))	Total effect (QALY (95% CI))	ICER (cost/QALY (95% CI))
Levofloxacin preventive therapy	$71.79 (44.21, 85.28)	17.59 (17.55, 17.60)	0.00
No levofloxacin preventive therapy	$94.94 (25.67, 113.51)	17.55 (17.41, 17.56)	−576.9 (242.2, −2893.5)
Difference per person (levofloxacin versus no levofloxacin)	−$23.15 (−57.87, −43.60)	0.04 (0.02, 0.18)	−576.9 (−242.2, −2893.5)
Difference per 1000 people (levofloxacin versus no levofloxacin)	−$23,145 (−5787; −43,604)	40.12 (20.2, 178.7)	−576.9 (−242.2, −2893.5)

ICER incremental cost-effectiveness ratio, MDR/RR-TB rifampicin resistant/multi-drug resistant tuberculosis, QALY quality adjusted life year, TPT tuberculosis preventative therapy, US United States.

**Fig. 2:**
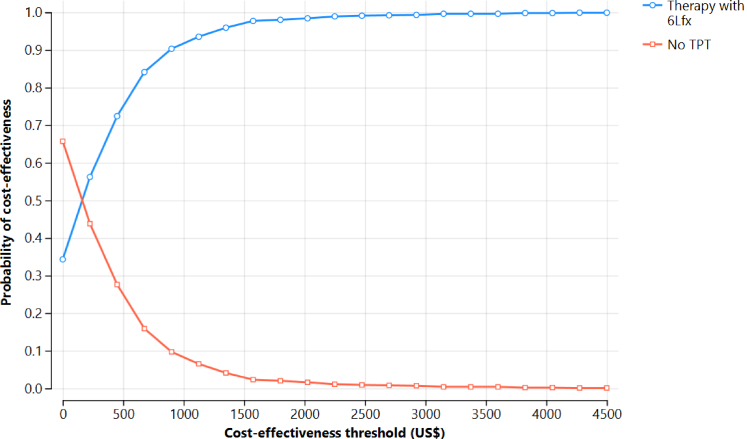
Cost effectiveness acceptability curve of 6Lfx versus no treatment for household contacts of MDR/RR-TB cases, over 1000 iterations.

**Fig. 3:**
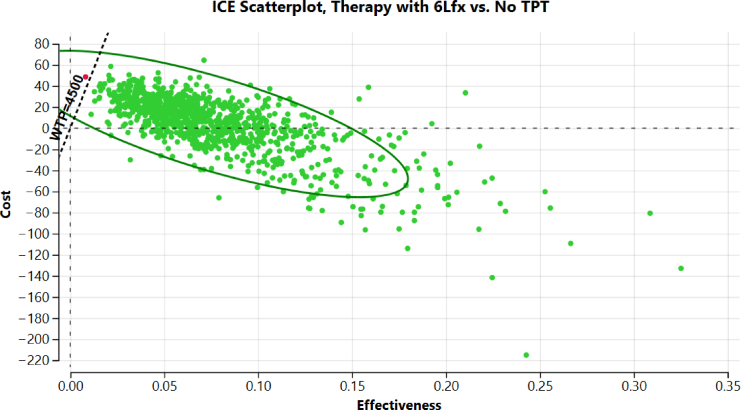
Cost effectiveness acceptability plane of 6Lfx versus not treatment for household contacts of MDR/RR-TB cases. 6Lfx 6 months of levofloxacin, ICE incremental cost effectiveness, TPT tuberculosis preventative therapy.

The probabilistic sensitivity analyses (Fig. 2, Fig. 3) found that the provision of 6Lfx to household contacts, compared to placebo, was a dominant strategy with high probability under a range of assumptions for model inputs. The cost-effectiveness acceptability curve (Fig. 2) confirmed cost-effectiveness over 1000 iterations (99.9% probability using a WTP of $4500).

### Sensitivity analyses

**Fig. 4:**
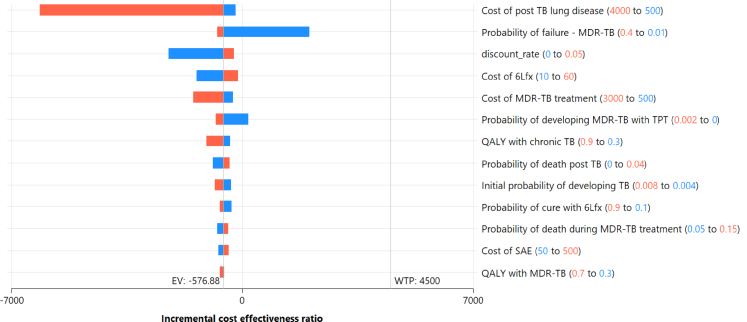
Tornado diagram showing effect of sensitivity analyses of the effect of variation of cost and event probability assumptions on the ICER for use of levofloxacin versus placebo in household contacts of people with MDR/RR-TB. 6Lfx 6 months of levofloxacin, ICER incremental cost effectiveness ratio, EV expected value, MDR-TB multi-drug resistant tuberculosis, SAE severe adverse event, TB tuberculosis, TPT tuberculosis preventative therapy, QALY quality adjusted life years, WTP willingness to pay.

The sensitivity analyses for variations in costs, utility and probabilities are shown in Fig. 4. Supplementary Table S2 presents the high- and low-cost and effectiveness values for the distributions used for each variable in the sensitivity analyses. The largest contributor to a change in ICER was the projected cost of post-TB lung disease averted. Despite the wide range in costs considered, the ICER remained below the WTP threshold. The cost of MDR/RR-TB treatment and the probability of failure of MDR/RR-TB treatment in secondary cases not averted were also important factors impacting the ICER, as was the cost of levofloxacin delivery, but all were well within the WTP threshold on sensitivity analyses. Overall, health system costs had the biggest impact on ICER.

## Discussion

This decision analysis model evaluated the cost-effectiveness of treating tuberculosis infection for six months with levofloxacin against no treatment in Vietnam a resource-limited setting. We applied a Markov model to compare the cost-effectiveness of a 6Lfx strategy for TB prevention, versus placebo, for household contacts of people diagnosed with MDR/RR-TB in Vietnam. We found that 6Lfx was both cost-saving and effective at preventing incident TB over a 20-year time horizon.

This is the first study assessing the cost-effectiveness of preventative therapy for MDR/RR-TB contacts in adults, using trial data. A previous study using a simulated model with fluroquinolone preventative therapy for MDR/RR-TB also found fluroquinolone therapy, in the United States, to be cost-effective if the effectiveness of therapy was greater than 10%.^23^ In the current study, the sensitivity analyses also found that 6Lfx is the dominant strategy if the lower bound of effectiveness was adjusted to 10%. This is despite the United States having higher treatment related costs, compared to Vietnam. The current study used trial data which may be a closer representation of real-life health care costs and QALYs, although it does not capture out-of-pocket expenses and other healthcare expenditures, which are difficult to quantify and were beyond the scope for the current analysis.

In these analyses, 40.1 QALYs were gained per 1000 treated individuals. No prior studies have evaluated the cost-effectiveness to preventive therapy for MDR/RR-TB in resource-limited settings. However, studies in which preventive therapy for DS-TB has been modelled have found similar values for QALYs saved per 1000 individuals.^24^, ^25^, ^26^ QALYs saved was found to be between 48.9 to 74.7^25^ per 1000 individuals compared to no treatment, and between 0.8 and 10 per 1000 individuals for rifapentine treatment strategies compared to isoniazid-based strategies.^24^^,^^25^ While two studies were in high income countries, one was in a refugee population in Turkey. This trial was conducted in both rural and urban provinces of Vietnam. While Vietnam has a national health insurance scheme, which ensures similar subsidies across the country, health costs may differ between these regions. Furthermore, since the study was conducted in just one country the findings of cost-effectiveness may not be generalisable to other settings. Further studies are required which consider the cost-effectiveness of TB preventive therapies in multiple settings with different costs and QALYs.

Overall, studies assessing the cost-effectiveness of preventative therapy for DS-TB have found most regimes to be cost effective and avert deaths compared to no treatment or compared to standard of care.^27^^,^^28^ The cost of treating TB disease is a significant driver in cost-effectiveness analysis. Despite this, newer more expensive DS-TB preventative strategies have also been shown to be cost effective,^24^^,^^26^ although the cheapest rifampicin-based regimes are the most cost-effective.^26^ These studies all provide evidence that preventing the development of TB is likely to be one of the factors to such interventions being cost-effective. The cost of levofloxacin is substantially cheaper than treatment for MDR/RR-TB as well as the long-term impact of post-TB lung disease, resulting in significant cost savings if TB disease can be prevented. In the current model, over 1000 iterations, the current model remained dominant, indicating that cost-effectiveness from a health system perspective persisted across large variances in uncertainty. The use of 6Lfx for treatment of TB is likely an important strategy in reducing TB prevalence in household contacts.

For most changes made in the sensitivity analyses, the ICER remained below the WTP threshold. In the present study, the incidence of grade 3 and 4 adverse events were similar in the two study arms, so varying their frequency did not have a substantial impact on the current Markov model. Varying the cost of 6Lfx treatment between US$10-$60 per course, changed the overall cost of the intervention arm between US$30-$80 per person. In other studies, the sensitivity analyses was found to be most affected by cost of the antibiotic used to treat TBI,^26^ increased risk of progression to TB,^23^^,^^24^^,^^29^^,^^30^ probability of cure^24^^,^^29^ and presence of adverse events.^25^

In the VQUIN trial 30.2% of individuals reported a Grade 1 or 2 adverse event, with 6.2% permanently discontinuing levofloxacin due to such an Grade 1 or 2 event.^6^ While these individuals likely experienced some reduction in their quality of life while taking treatment, their symptoms were generally brief and self-limiting. However, as they were not hospitalised they did not incur significant healthcare costs. Therefore, as our analysis was from the health system perspective, costs associated with low-grade adverse events was not incorporated in our estimates of cost-effectiveness or quality of life. Further research is required to evaluate the economic impacts of poorly-tolerated TPT in Vietnam and other settings.

There is growing evidence that over half of TB survivors have impaired lung function after successful treatment of TB.^18^ However, the exact burden of disease, extent of respiratory disease and costs associated is poorly defined. The model described in the current study remained cost-effective despite using a wide interval for both the cost (between US$500 to US$4000), and QALY (0.3–0.9) estimates for post-TB lung disease. There is limited existing literature which incorporates post-TB lung disease into current cost-effectiveness analysis. Only one study made an estimate for QALY in TB survivors in their model,^24^ but no studies incorporated cost-estimates. While estimates used in the current study is based on literature which includes health care costs and losses to income, there is limited precision in the estimates of post-TB lung disease and further research is required to determine the effect of MDR-TB upon long-term healthcare utilisation and quality of life.

The sensitivity analysis also included wide intervals for the cost of MDR/RR-TB. While the Vietnamese NTP is transitioning over to the BPaL/M for MDR/RR-TB, it is not always widely available, and the costs can be significant. Hence, we used a wide range for the cost of MDR/RR-TB in the sensitivity analysis, between US$500 and US$3000, to account for differences in drug procurement costs as well as costs of different regimens. Furthermore, the sensitivity analysis also incorporated a wide range for MDR/RR-TB associated mortality. Despite these wide intervals, the model remained cost-saving which ultimately underlines the importance of the prevention of TB itself to remain cost-effective and also prevent the health impacts of TB upon the community.

Strengths of this study included the use of trial-based values for effectiveness. We also measured health system costs within the trial to improve application within real life contexts. A further strength was the inclusion of a sensitivity analyses, with wide upper and lower bounds for each variable. Despite this, the ICER remained below the WTP for most variables. The model remained cost-effective across all ranges. Furthermore, the probability of cure used in the model was based on Bayesian analysis of two randomised control trials, providing a more precise value for effectiveness of 6Lfx therapy.

These analyses have several limitations. While the sensitivity analyses provide confidence that the model remains cost-effective despite wide ranges in costs, there are several variables with considerable uncertainty around the cost estimates. These included the cost MDR/RR-TB treatment, and post-TB lung disease averted, given the limited published data on the cost of newer short-course MDR/RR-TB treatment regimens and the cost associated with post-TB lung disease case in resource-limited settings. No published estimates for Vietnam were available. While age adjusted mortality and age-adjusted QALY have been factored into the model, age-specific cost-data were not available. Therefore, our findings may over-estimate or under-estimate cost-effectiveness for certain age-groups.

Despite the fact that VQUIN is a large RCT undertaken for TPT in contacts of people with MDR/RR-TB, the transition probabilities that informed this model are based on relatively small numbers of people with incident TB during the trial follow-up, limiting confidence in its generalisability, with potential differences during ‘real life’ implementation likely to affect the cost-effectiveness estimates. The model also excludes the possibility of reinfection disease, which is a real concern in high TB incidence settings with uncontrolled MDR/RR-TB transmission. This may underestimate the true incidence of TB and overestimate the QALY’s saved with treatment with 6Lfx. Finally, the treatment outcomes with newer MDR/RR-TB in operational settings is yet to be determined and may change with newly emerging drug resistance. As better data becomes available for these regimes and variable drug resistance patterns in different setting, the cost-effectiveness of 6Lfx may be impacted.

Ideally, the implementation of the 6Lfx strategy should incorporate further collection of longitudinal data including the long-term effectiveness of TPT and more rigorous cost estimates from programmatic settings. Future studies and data about the impacts to patient related cost and quality of life from TB and post-TB lung disease are also required.

In conclusion, the best available evidence indicates that 6Lfx for treatment of MDR/RR-TB household contacts MDR/RR-TB is highly cost-effective in high TB incidence settings. In this setting, the intervention was associated with lower costs and greater quality of life.” These findings have important policy implications. WHO has strongly recommended levofloxacin preventative therapy is an important strategy to reduce secondary TB cases,^31^ improving the health of infected individuals and preventing ongoing transmission of drug-resistant *M. tuberculosis*.

## Contributors

TH data curation, data analysis, manuscript review.

NTA data curation, study design, manuscript review.

NBH manuscript review.

NVN manuscript review.

MY data curation, manuscript review.

SG study design, manuscript review.

BM study design, manuscript review.

GM study design, manuscript review.

TL supervision, study design, data analysis, manuscript review.

GF supervision, study design, manuscript review.

TH and TL accessed and verified the data.

TH and GF have the final responsibility to submit this manuscript.

## Data sharing statement

Data that supports the findings of this study are available from the corresponding author upon request.

## Declaration of interests

GM received a grant from the National Health and Medical Research Council which supported this work. He is also the President, International Union Against TB and Lung Disease.

GF is a member of the data safety and monitoring board of the DRAMATIC trial. He is also a board member of the Australian Respiratory Council. GF has received budesonide/formoterol from Astra-Zeneca for a clinical trial of asthma and COPD.

NVN received a grant from the National Health and Medical Research Council which supported this work (grant #1081443).

TAN received a grant from the National Health and Medical Research Council which supported this work (grant #1081443).
