# Supplementary material for: Evaluating the cost-effectiveness of levofloxacin therapy for household contacts of multidrug-resistant tuberculosis in Vietnam
Source: Lancet Reg Health West Pac. 2025 Aug 8;61:101666. doi: 10.1016/j.lanwpc.2025.101666 (PMC12355538; doi:10.1016/j.lanwpc.2025.101666)
Supplement: Supplementary Figs. S1 and S2, Tables S1 and S2 [file mmc1.docx]

**Supplement Figure 1: The Markov decision tree**


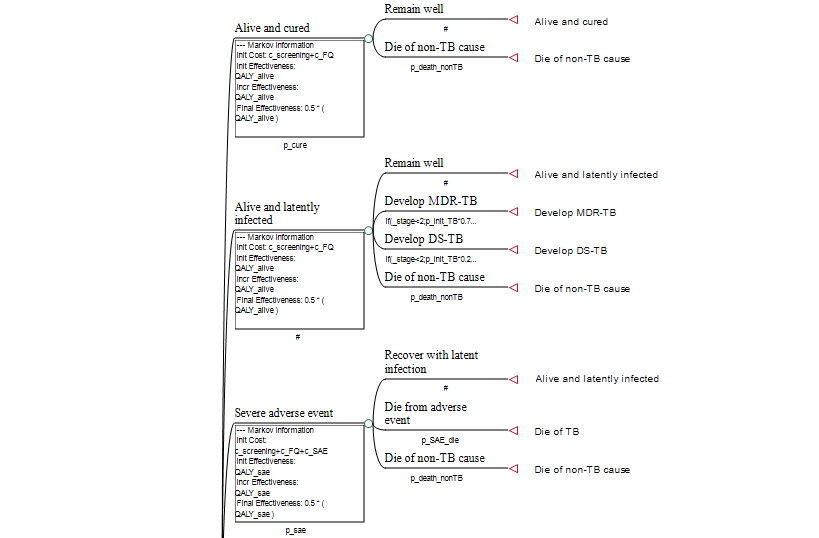


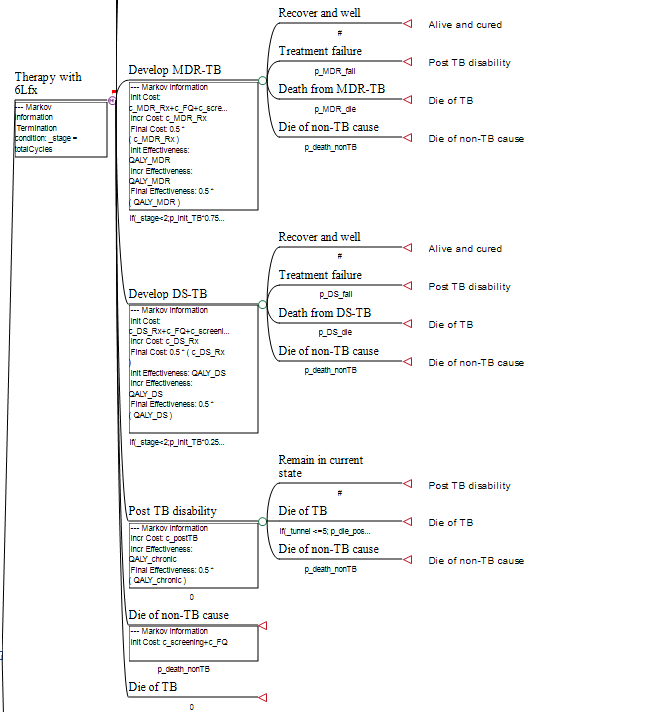


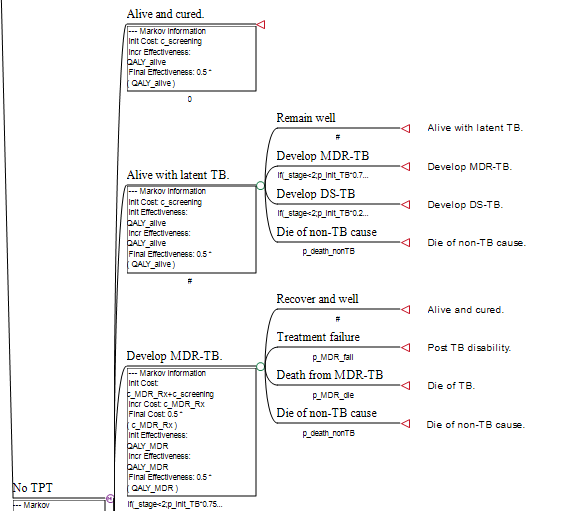


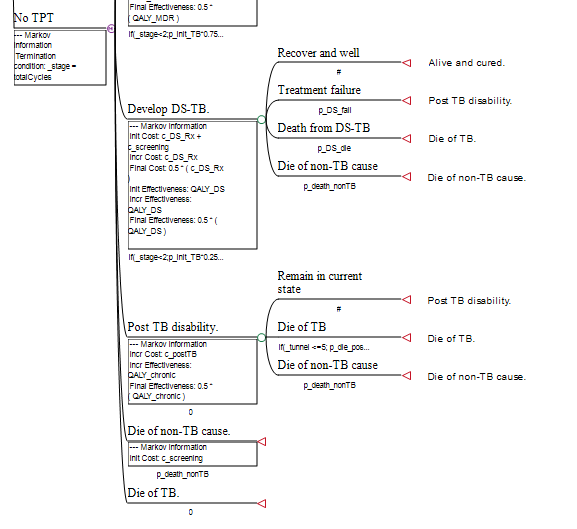


**Supplement Figure 2: The Markov decision model**


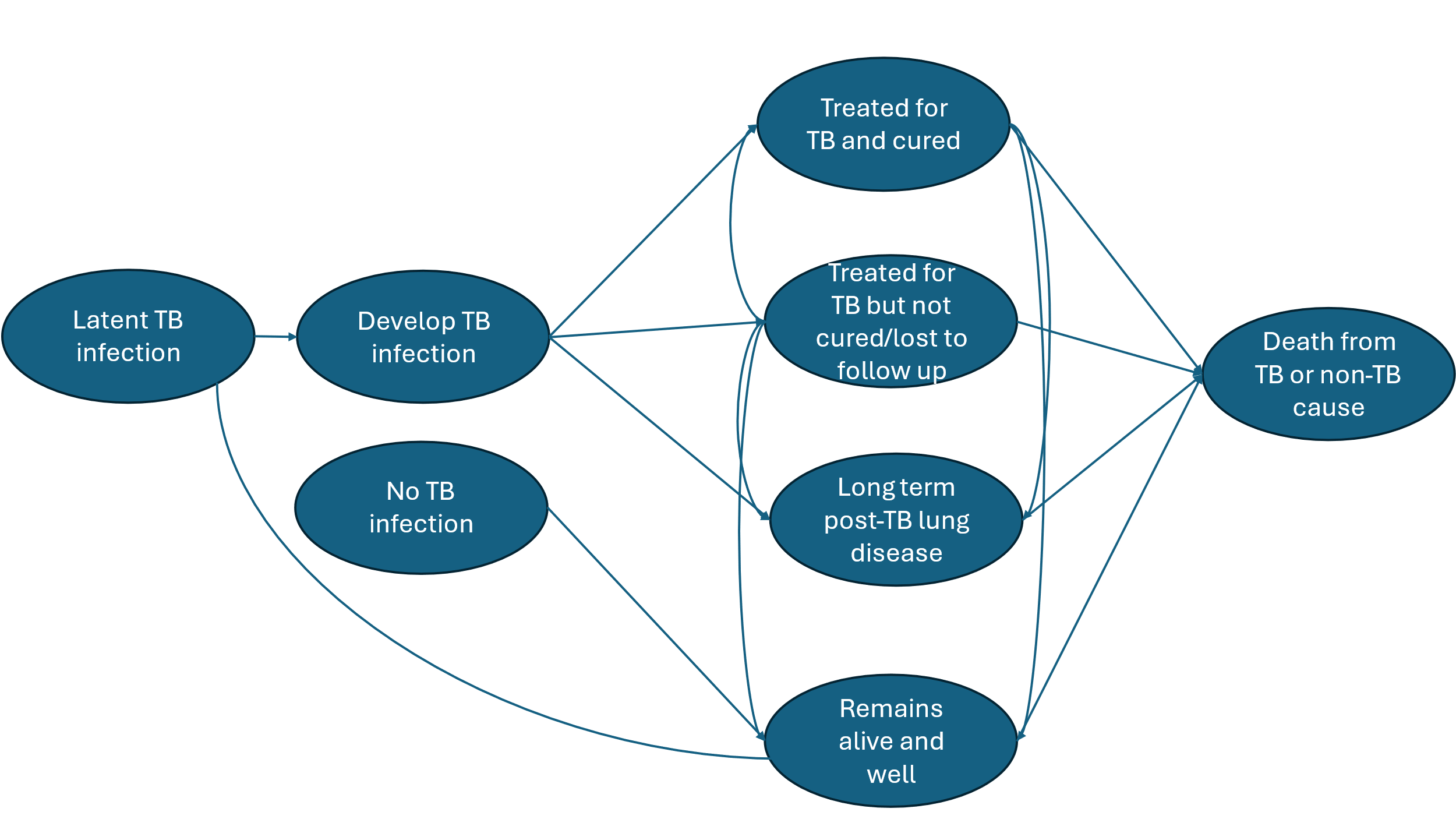


**Table S2: Sensitivity analyses: Impact on cost and effectiveness**

| **Variable Description** | **Low estimate** | **High estimate** | **Low estimate for effectiveness** | **High estimate for effectiveness** | **Low estimate for cost saving ($US)** | **High estimate for cost saving ($US)** |
| --- | --- | --- | --- | --- | --- | --- |
| Initial probability of developing TB | 0ꞏ004 | 0ꞏ008 | 17ꞏ582 | 17ꞏ590 | 67ꞏ72 | 78ꞏ57 |
| Probability of cure with 6Lfx | 0ꞏ2 | 0ꞏ8 | 17ꞏ577 | 17ꞏ592 | 65ꞏ97 | 83ꞏ44 |
| Probability of death during DS-TB | 0ꞏ005 | 0ꞏ04 | 17ꞏ587 | 17ꞏ587 | 71ꞏ79 | 71ꞏ79 |
| Probability of death during RR/MDR-TB | 0ꞏ05 | 0ꞏ15 | 17ꞏ583 | 17ꞏ590 | 71ꞏ79 | 71ꞏ79 |
| Probability of death post TB | 0 | 0ꞏ04 | 17ꞏ586 | 17ꞏ589 | 70ꞏ06 | 73ꞏ89 |
| Probability of failure - DS-TB | 0ꞏ01 | 0ꞏ1 | 17ꞏ587 | 17ꞏ588 | 70ꞏ95 | 72ꞏ21 |
| Probability of failure – RR/MDR-TB | 0ꞏ01 | 0ꞏ4 | 17ꞏ585 | 17ꞏ594 | 36ꞏ66 | 75ꞏ60 |
| Probability of developing RR/MDR-TB with TPT | 0 | 0ꞏ002 | 17ꞏ583 | 17ꞏ592 | 63ꞏ26 | 76ꞏ86 |
| Probability of Grade 3-5 adverse | 0 | 0ꞏ05 | 17ꞏ578 | 17ꞏ598 | 64ꞏ74 | 77ꞏ33 |
| Discount rate | 0 | 0ꞏ05 | 17ꞏ584 | 17ꞏ592 | 69ꞏ03 | 77ꞏ57 |
| Cost of 6Lfx ($US) | 10 | 60 |  |  | 39ꞏ79 | 89ꞏ79 |
| Cost of SAE ($US) | 50 | 500 |  |  | 65ꞏ91 | 78ꞏ51 |
| Cost of screening ($US) | 0 | 4 |  |  | 69ꞏ39 | 73ꞏ39 |
| Cost of RR/MDR-TB treatment ($US) | 500 | 3000 |  |  | 68ꞏ43 | 80ꞏ63 |
| Cost of DS-TB treatment ($US) | 50 | 200 |  |  | 71ꞏ67 | 71ꞏ91 |
| Cost of post TB lung disease ($US) | 500 | 4000 |  |  | 67ꞏ21 | 133ꞏ89 |
| QALY with RR/MDR-TB | 0ꞏ3 | 0ꞏ9 | 17ꞏ580 | 17ꞏ592 |  |  |
| QALY with DS-TB | 0ꞏ3 | 0ꞏ7 | 17ꞏ586 | 17ꞏ588 |  |  |
| QALY with chronic TB | 0ꞏ5 | 0ꞏ9 | 17ꞏ587 | 17ꞏ588 |  |  |

6Lfx 6 months of levofloxacin, DS-TB drug susceptible tuberculosis, QALY Quality adjusted life years, RR/MDR-TB rifampicin resistant/multi-drug resistant tuberculosis, SAE severe adverse event, TB tuberculosis, TPT TB preventative therapy, US United States
